# Supplementary material for: Genomic Divergence in Swedish Warmblood Horses Selected for Equestrian Disciplines
Source: Genes (Basel). 2019 Nov 27;10(12):976. doi: 10.3390/genes10120976 (PMC6947233; doi:10.3390/genes10120976)
Supplement: Supplementary file 1 [file genes-10-00976-s001.zip › Supplementary Figure 1.pdf]

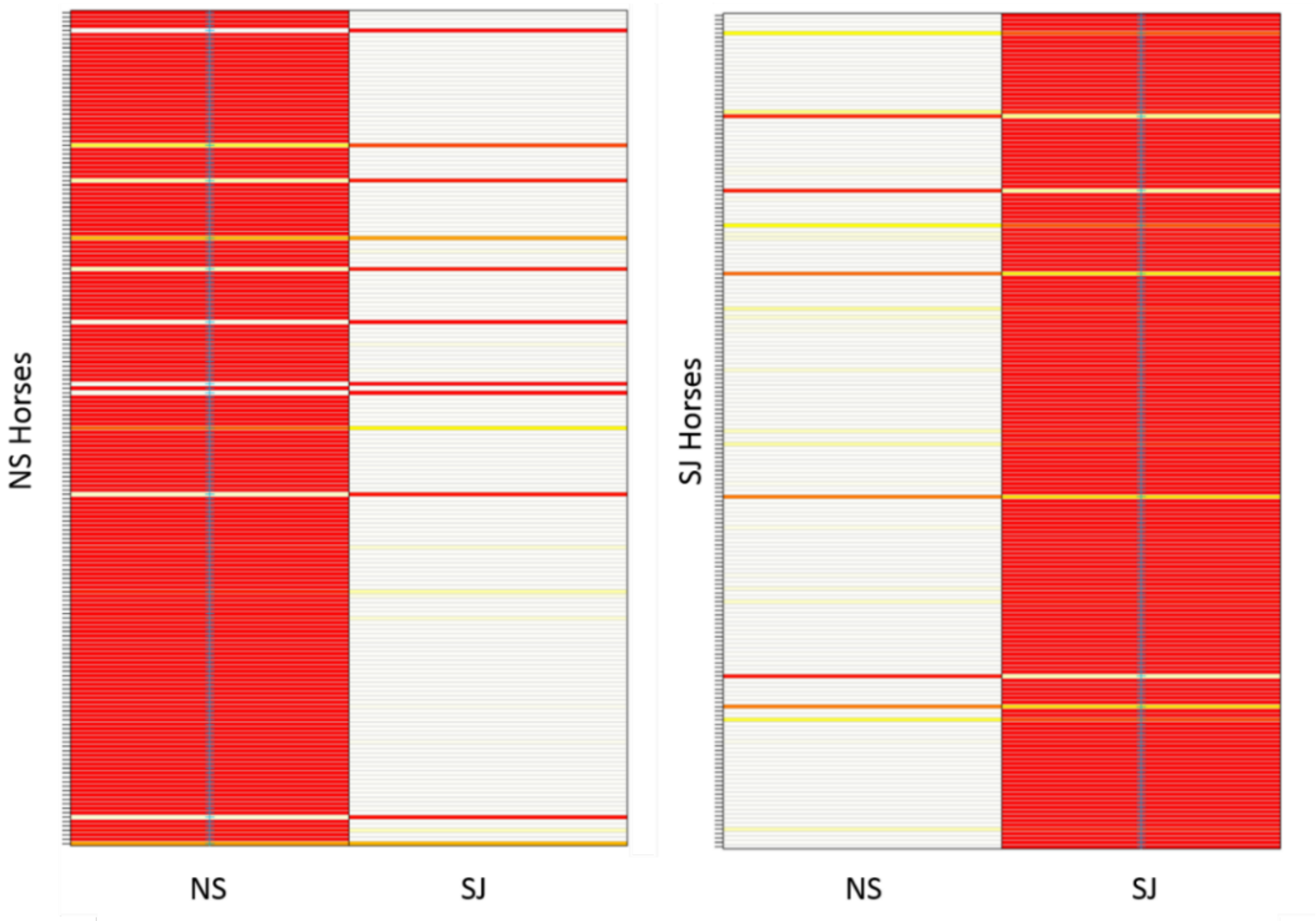

Supplementary Figure 1. Heatmap showing the posterior probability of each individual being assigned to the predefined subpopulations based on EBVs where non-show jumping horses (NS) are shown in the left panel and show jumping horses (SJ) are shown in the right panel. A red bar represents a membership probability equal to one and a white bar represents a membership probability of zero.
